# Iron distribution in different tissues of homozygous Mask (msk/msk) mice and the effects of oral iron treatments

**DOI:** 10.1002/ajh.26311

**Published:** 2021-08-14

**Authors:** Michela Asperti, Elisa Brilli, Andrea Denardo, Magdalena Gryzik, Francesca Pagani, Fabiana Busti, Germano Tarantino, Paolo Arosio, Domenico Girelli, Maura Poli

**Affiliations:** ^1^ Department of Molecular and Translational Medicine University of Brescia Brescia Italy; ^2^ Pharmanutra S.p.a. Pisa Italy; ^3^ Department of Medicine University of Verona Verona Italy; ^4^ Azienda Ospedaliera Integrata Verona Veneto Region Referral Center for Iron Metabolism Disorders, GIMFer (Gruppo Interdisciplinare sulle Malattie del Ferro) Verona Italy

## Abstract

Iron‐refractory iron deficiency anemia (IRIDA) is an autosomal recessive disorder caused by genetic mutations on *TMPRSS6* gene which encodes Matriptase2 (MT2). An altered MT2 cannot appropriately suppress hepatic BMP6/SMAD signaling in case of low iron, hence hepcidin excess blocks dietary iron absorption, leading to a form of anemia resistant to oral iron supplementation. In this study, using the IRIDA mouse model Mask, we characterized homozygous (msk/msk) compared to asymptomatic heterozygous (msk/wt) mice, assessing the major parameters of iron status in different organs, at different ages in both sexes. The effect of carbonyl iron diet was analyzed as control iron supplementation being used for many studies in mice. It resulted effective in both anemic control and msk/msk mice, as expected, even if there is no information about its mechanism of absorption. Then, we mainly compared two forms of oral iron supplement, largely used for humans: ferrous sulfate and Sucrosomial iron. In anemic control mice, the two oral formulations corrected hemoglobin levels from 11.40 ± 0.60 to 15.38 ± 1.71 g/dl in 2–4 weeks. Interestingly, in msk/msk mice, ferrous sulfate did not increase hemoglobin likely due to ferroportin/hepcidin‐dependent absorption, whereas Sucrosomial iron increased it from 11.50 ± 0.60 to 13.53 ± 0.64 g/dl mainly in the first week followed by a minor increase at 4 weeks with a stable level of 13.30 ± 0.80 g/dl, probably because of alternative absorption. Thus, Sucrosomial iron, already used in other conditions of iron deficiency, may represent a promising option for oral iron supplementation in IRIDA patients.

## 1 INTRODUCTION

Hepcidin is the main regulator of systemic iron homeostasis reducing iron absorption, its recycling and mobilization from stores by the promotion of ferroportin (FPN) internalization and degradation.^1^, ^2^ Liver hepcidin expression is regulated by various factors, mostly connected with iron availability mainly by bone morphogenetic protein 6 (BMP6), inflammation by Interleukin 6 (IL6), hypoxia by hypoxia‐inducible factor 2α (HIF2α), and erythropoietic activity by Erythroferrone (ERFE).^3^, ^4^, ^5^

Note, BMP6, produced by liver non‐parenchymal cells,^6^, ^7^, ^8^ interacts with its receptors and the co‐receptor hemojuvelin (HJV), participating in BMP/SMAD1/5/8 signaling. Other BMPs might be also potentially involved in hepcidin induction such as BMP2.^9^, ^10^ Hemojuvelin supports the activation of the signalling^11^ and its degradation, carried out by the serine protease matriptase2 (MT2), guarantees the control of hepcidin levels.^12^ Recently it has been reported that MT2 cleaves also BMP receptors (BMPR) ALK2, ALK3, ActRIIa, BMPR2, the homeostatic iron regulator protein (HFE) and transferrin receptor 2 (Tfr2)^13^ on the cell membrane, revealing a complex function. On the other hand, also the ectodomain of MT2 seems to play an important nonproteolytic role in suppressing hepcidin expression in mice.^14^ It suggests that the ectodomain of MT2 is sufficient to limit the induction of hepcidin expression or it is involved in the formation of the complex that, in absence of the proteolytic activity, it could sustain the activation of the complex in vivo, but more studies are essential to shed light on the complex role of MT2 in the regulation of hepcidin expression.

Mutations of the *TMPRSS6* gene, that causes defective MT2 at the level of proteolytic domain making the protein not able to appropriately process HJV, lead to a hepcidin excess and an anemia refractory to oral iron treatments named iron Refractory Iron Deficiency Anemia (IRIDA).^15^, ^16^, ^17^ The classical features of IRIDA patients are: increased plasma hepcidin levels,^18^ moderate/severe anemia (Hemoglobin (Hb) 6–9 g/dl), hypochromic, microcytic anemia (MCV 45–65 fL), low transferrin saturation (<5%), normal or even moderately elevated serum ferritin level, normal C‐reactive protein.^19^, ^20^, ^21^ The current treatment is mainly based on parenteral iron supplementation which usually improves but does not completely correct anemia, making the control of hepcidin expression or new iron formulations promising approaches to solve the anemia. Ferrous sulfate is the iron (II) salt of sulfuric acid, effectively used in humans to treat iron deficiency in several conditions. Its absorption is presumably dependent on the divalent metal transporter 1 (DMT1)/FPN/hepcidin axis, which explains its poor effectiveness in anemic patients characterized by high hepcidin levels.

Interestingly, Capra et al.^22^ recently published a case study on a child with IRIDA observing a gradual increase in Hb level after the oral treatment with Sucrosomial iron.^22^ It is based on ferric pyrophosphate covered by a phospholipid plus sucrose esters of fatty acids matrix. Experiments using cultured CaCo‐2 cells, showed that it is readily absorbed, possibly by a mechanism not involving the canonical transporter DMT1,^23^ but further studies are necessary to fully clarify its mechanism of absorption. Noteworthy, Sucrosomial iron has been reported to be effective also for iron deficiency in celiac disease,^24^ post‐bariatric surgery anemia,^25^ myelodysplastic syndromes,^26^ inflammatory bowel disease^27^ and cancer.^28^, ^29^

In our recent work,^30^ we showed that healthy mice are not able to absorb oral iron in the forms of both ferrous sulfate and Sucrosomial iron. On the other hand, when the same mice become anemic by iron‐low diet (Hb about 11 to 12 g/dl), they readily absorb both the iron formulations solving the anemia.

It is also important to study these two oral iron formulations in a high‐hepcidin disorder. There are three available mouse models of IRIDA, showing similar phenotypes with truncal alopecia, iron‐deficiency anemia and elevated expression of hepcidin.^15^, ^16^, ^17^ They have been treated with intraperitoneal injections (IP) iron‐dextran,^16^ carbonyl iron‐rich diet, Erythropoietin (EPO),^31^ anti‐HJV antibody,^32^ inhibitors of BMP6 pathway,^33^ or crossed with *HJV* KO or *HFE* KO mice.^34^, ^35^ Anemia was rescued by 1 mg/g iron‐dextran (Hb from 8 to 12 g/dl) or by 2% carbonyl iron diet (Hb from 9 to 11.5 g/dl)^16^ for 3 weeks. Whereas, EPO treatment has failed both to downregulate hepcidin and to solve the anemia.^31^

In the present work, we studied the homozygous (msk/msk) and heterozygous (msk/wt) Mask mouse strain^16^ (males and females) from 3 to 28 weeks of age, by following iron content in several tissues, body weight, hepcidin, serum iron, hemoglobin (Hb), hematocrit (Ht) and some markers of BMP6/SMAD pathway and inflammation. This allowed to select the 9‐weeks old mice (when the main hematological and iron parameters were stabilized) as the best period to evaluate the effects of Ferrous sulfate and Sucrosomial iron, in order to investigate possible differences in iron absorption and to identify the best strategy to treat IRIDA disease.

## 2 METHODS

### 2.1 Animals

The C57BL/6J‐Tmprss6^msk^/Mmucd mice (number 016987‐UCD) were obtained from MMRRC, a NCRR‐NIH funded strain repository.^16^ All the procedures were approved by the Animal Care and Use Committee of University of Brescia. Hematological parameters (Hb and Ht) analyzed using Hemo_Vet Instrument (Infratech) by collecting a single drop of blood and weight were monitored weekly in the morning.

### 2.2 Treatments

#### 2.2.1 Iron rich diet

The msk/msk or msk/wt mice (9‐weeks old mice, both males and females, four mice per group) were kept in iron balance (200 mg/kg of pellets carbonyl‐iron, Scientific Animal Food & Engineering, SAFE) or on iron‐rich diet (8.3 g/kg of pellets carbonyl‐iron, SAFE). After 10 days, Hb and Ht were analyzed, then mice were sacrificed and tissues collected for analysis.

#### 2.2.2 Ferrous sulfate and Sucrosomial iron treatment

All msk/msk mice (9‐weeks old female mice, four mice per group) were kept at an iron balance diet and treated daily with 0.5 or 4 mg of elemental iron per kg/body weight contained in ferrous sulfate and Sucrosomial iron (patent n° PCT/IB2013/001659 owned by Alesco s.r.l, Italy) or vehicle (same composition of Sucrosomial iron but without pyrophosphate iron^30^) by gavage for 35 days. In the experiment with msk/wt mice (3‐weeks old female mice) were kept on an iron‐free diet (about 5 mg/kg of pellets carbonyl‐iron, SAFE) for 6 weeks and one group (four mice) was maintained in iron balance diet as a control. The treatments started when the Hb fell below 12.5 g/dl (after about 6 weeks). The mice were daily treated with vehicle, ferrous sulfate and Sucrosomial Iron, by oral‐gavage, for 28 days, and Hb and Ht were monitored weekly.

### 2.3 Analysis

Mice were sacrificed at the indicated time points. Blood and different tissues were collected for analysis of mRNA expression; iron content in tissues and serum; hepcidin, erythropoietin (EPO), erythroferrone (ERFE) in serum; peripheral blood smear staining; histochemistry and immunostaining on paraffin‐embedded duodenal sections. The detailed procedures are reported in Appendix S1.

### 2.4 Statistics

Comparison between heterozygous and homozygous mice matched for age and sex were performed by two‐way ANOVA. Multiple comparisons were corrected by Sidak's test. For treatments, comparisons between vehicle versus iron formulations were performed using One‐way ANOVA with multiple comparisons corrected by Tukey's test. Data are shown as dot plot, with Mean ± SD.

## 3 RESULTS

### 3.1 Hematological parameters and iron status in msk/msk mice

**FIGURE 1:**
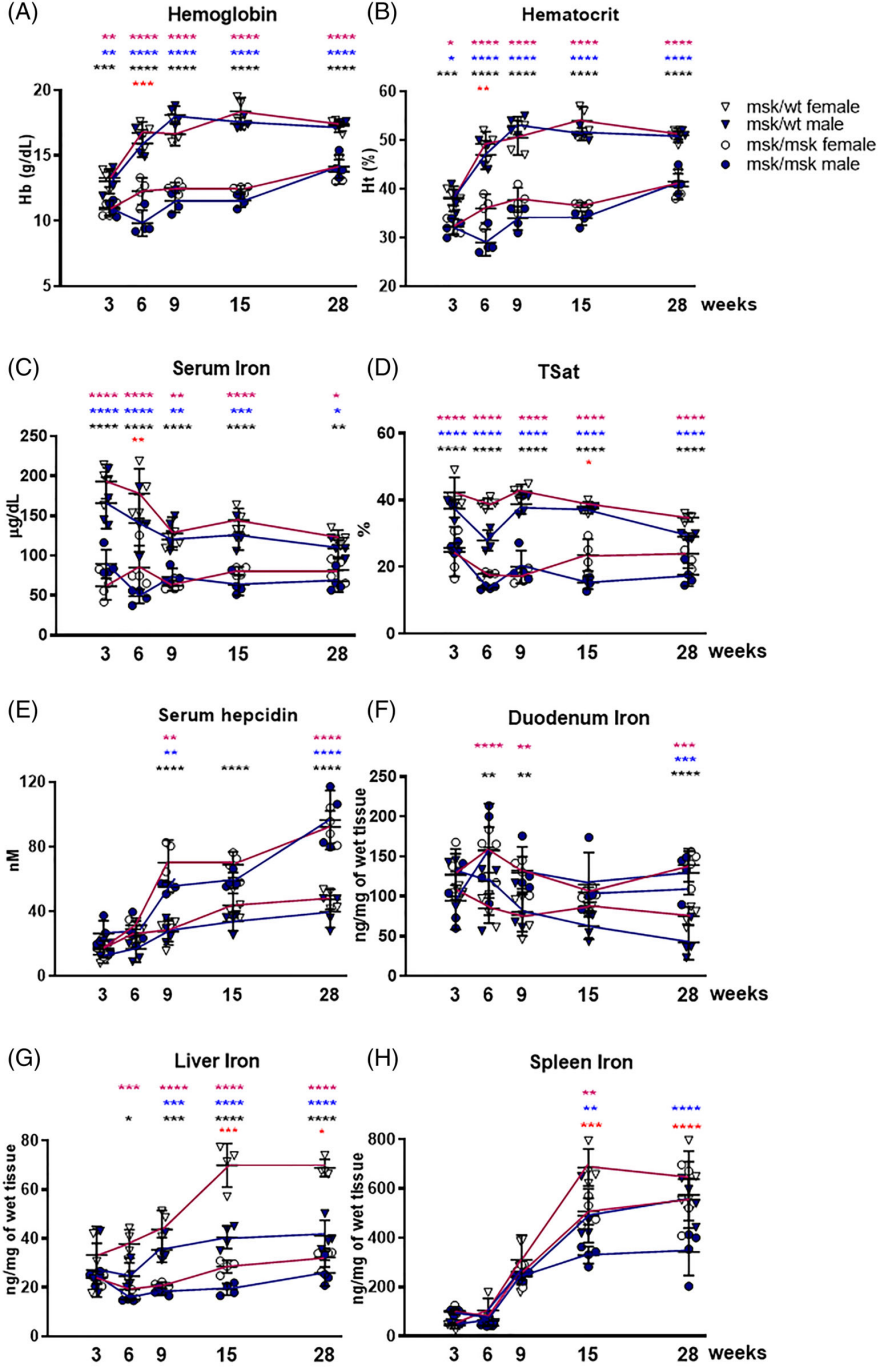
Iron and hematological parameters in msk/msk and msk/wt female and male mice in different ages. **(**A) Hemoglobin (Hb) and (B) Hematocrit (Ht) levels measured using Hemo_Vet instrument; (C) Serum iron and (D) Transferrin Saturation (TSat) were measured using commercial kit; (E) Hepcidin protein in serum was measured by Surface Enhanced Laser Desorption Ionization Time of Flight‐Mass Spectrometry (SELDI‐TOF). (F) Duodenum, (G) Liver and (H) Spleen iron content was detected spectrophotometrically. The female mice were marked with pink line and empty circles (homozygous, msk/msk) or inverted triangles (heterozygous, msk/wt), whereas male in blue line and blue circles (homozygous, msk/msk) or inverted triangles (heterozygous, msk/wt). Each group consisted of four animals. Statistical analysis comparison between: msk/msk and msk/wt female for each age (pink asterisks); msk/msk and msk/wt male (blue asterisks); msk/msk and msk/wt mixed sexes each age (black asterisks); msk/msk female and msk/msk male for each age (red asterisks). ****P < 0.0001, ***p < 0.001, **p < 0.01, *p < 0.05 [Color figure can be viewed at wileyonlinelibrary.com]

The Mask mice^16^ recapitulate the human IRIDA hematological phenotype. It is known that they are characterized by alopecia in the trunk with the hair only in the face and they are smaller than wild‐type (wt/wt) and msk/wt mice as observed, throughout the entire period of 28 weeks, with a 2–6 grams lower body weight (Table S1). The msk/msk mice had hepcidin levels two‐to‐three‐fold higher than those of msk/wt and wt/wt mice (Figure 1(E)). Due to the high level of hepcidin, the msk/msk mice were anemic at all the ages tested compared to the msk/wt mice that showed Hb and Ht values similar to wt/wt mice (Figure 1(A),(B) and Table S1), making the msk/wt mice as feasible controls of the msk/msk mice. As reported in the Table S2, the msk/msk showed lower MCV compared to msk/wt (30.36 ± 3.58 fL vs. 44.73 ± 1.80 fL) indicating a microcytic anemia; in addition, the level of the reticulocyte hemoglobin content (Ret‐He) was reduced in all the ages analyzed (3–9 to 15–28 weeks) of msk/msk male and female mice (11.21 ± 0.80 pg vs. 16.65 ± 0.82 pg).

Specifically, at 3 weeks the Hb and Ht values were similar in msk/msk and control msk/wt mice, the difference among them became apparent with age particularly at 9–15 weeks. The msk/wt mice showed a fast increase of Hb levels between 3 and 6 weeks, and then it leveled off at 17.54 ± 0.84 g/dl, in both sexes. The msk/msk mice showed a steady but minor increase in Hb levels up to 28 weeks, to reach a plateau value of 13.95 ± 0.87 g/dl, with similar kinetics in the females and in males, making a significant difference between them only at 6 weeks (Figure 1(A)). The Ht levels showed a similar trend to Hb (Figure 1(B) and Table S1). As expected, liver hepcidin mRNA showed a pattern opposite to that of Hb, with a fast three‐fold increase in msk/msk mice between 3 and 6 weeks compared with a minor increase in the msk/wt controls. The difference increased with age, but without evident differences between the sexes in both mice strains (Figure S1(A)). Serum hepcidin levels showed similar but slower behavior, with a major increase of four‐fold in the msk/msk mice between 3 and 9 weeks, higher in females than males, while in the msk/wt mice the level remained rather steady during the whole period of analysis (Figure 1(E)).

The Id1 mRNA showed the same trend of hepcidin (Figure S1(B)). Liver SOCS3 mRNA level showed similar values in msk/msk and msk/wt mice without differences between the sexes, indicating no signs of inflammation (Figure S1(C)).

Serum iron strongly decreased from 179.50 ± 29.30 to 123.94 ± 15.34 μg/dl in the msk/wt mice at 3–9 weeks that corresponds to the period of Hb increase, then it leveled off at 125.24 ± 17.95 μg/dl at 15–28 weeks. In the msk/msk mice the serum iron remained remarkably stable at 71.25 ± 17.91 μg/dl during the whole period, and always lower than that of the control mice (Figure 1(C)) with a similar behavior for TSat (Figure 1(D)). The opposite was detected in the duodenum in which msk/msk mice showed higher iron levels than the msk/wt mice (128.68 ± 34.73 ng/mg vs. 86.06 ± 31.49 ng/mg of wet tissue respectively in both sexes) despite a large intra‐group variability (Figure 1(F)). The msk/msk mice had liver iron content significantly lower than that of msk/wt mice at all the ages analyzed, apart from at 3 weeks in which there was high variability (Figure 1(G)). The differences between msk/msk and msk/wt mice became more pronounced with age and more evident in females since that Mask ones had rather stable values of 24.93 ± 5.60 ng/mg of wet tissue while the msk/wt increased the liver iron content with an age‐dependent trend from 38.20 ± 9.14 ng/mg (at 3–6‐9 weeks) to 69.39 ± 6.28 ng/mg (at 15–28 weeks) (Figure 1(G)). The spleen iron content was similar in msk/msk and msk/wt mice until 9 weeks, then the spleen iron content decreased in msk/msk mice in both sexes (Figure 1(H)). Females msk/wt mice showed higher spleen and liver iron than males msk/wt, in agreement with those described for C57BL/6J strain.^36^, ^37^

The effect of hepcidin on the iron status of tissues is unclear, and the Mask mice offer the opportunity to elucidate it. In the brain, the iron content showed a tendency to increase with age in both sexes and maintained a significant difference between the two mice strains, ranging from 6.72 ± 0.62 to 9.33 ± 0.65 ng/mg in the msk/msk mice and from 7.02 ± 0.94 to 13.16 ± 1.07 ng/mg in msk/wt mice (Figure S2(A)). Similarly, in the heart and kidney, the iron content showed a tendency to be lower in the Mask than in control mice in both sexes (Figure S2(B),(C)). In the heart the iron content varied in the first period and then it leveled off at 9 weeks without strong differences between the sexes (Figure S2(B)). The iron content stabilized also in the kidney after 9 weeks, and the control msk/wt females showed a higher iron content than the males (Figure S2(C)). Gastrocnemius and vastus lateralis muscles (Figure S2(D),(E)) (both involved in locomotion but differing in twitch fibers content, about 50% in the gastrocnemius and 32% in the vastus lateralis), showed a reduced iron content in Mask both in females and males during the ages, but more evident in the gastrocnemius, with the lowest level at 9 weeks, suggesting this as the best period to compare the muscle performances of the two mice. In summary the Mask mice showed a lower iron content than the control heterozygous mice in all the organs analyzed, except for the duodenum.

### 3.2 Iron treatments

The above data showed that in the Mask mice, anemia was maximal during the 9–15 weeks period in correspondence of the highest serum hepcidin levels (maintained till about 28 weeks), suggesting this as the best interval of time for studying the effects of iron treatments.

The high levels of hepcidin are expected to inhibit intestinal iron absorption by inducing FPN degradation, making anemia refractory to oral iron supplementation. However, it was shown that when Mask mice receive an iron‐rich diet (2% of carbonyl iron) for 1 to 3 weeks, anemia is partially reverted with an increase of Hb from about 8 to 9–12 g/dl, respectively.^16^

**FIGURE 2:**
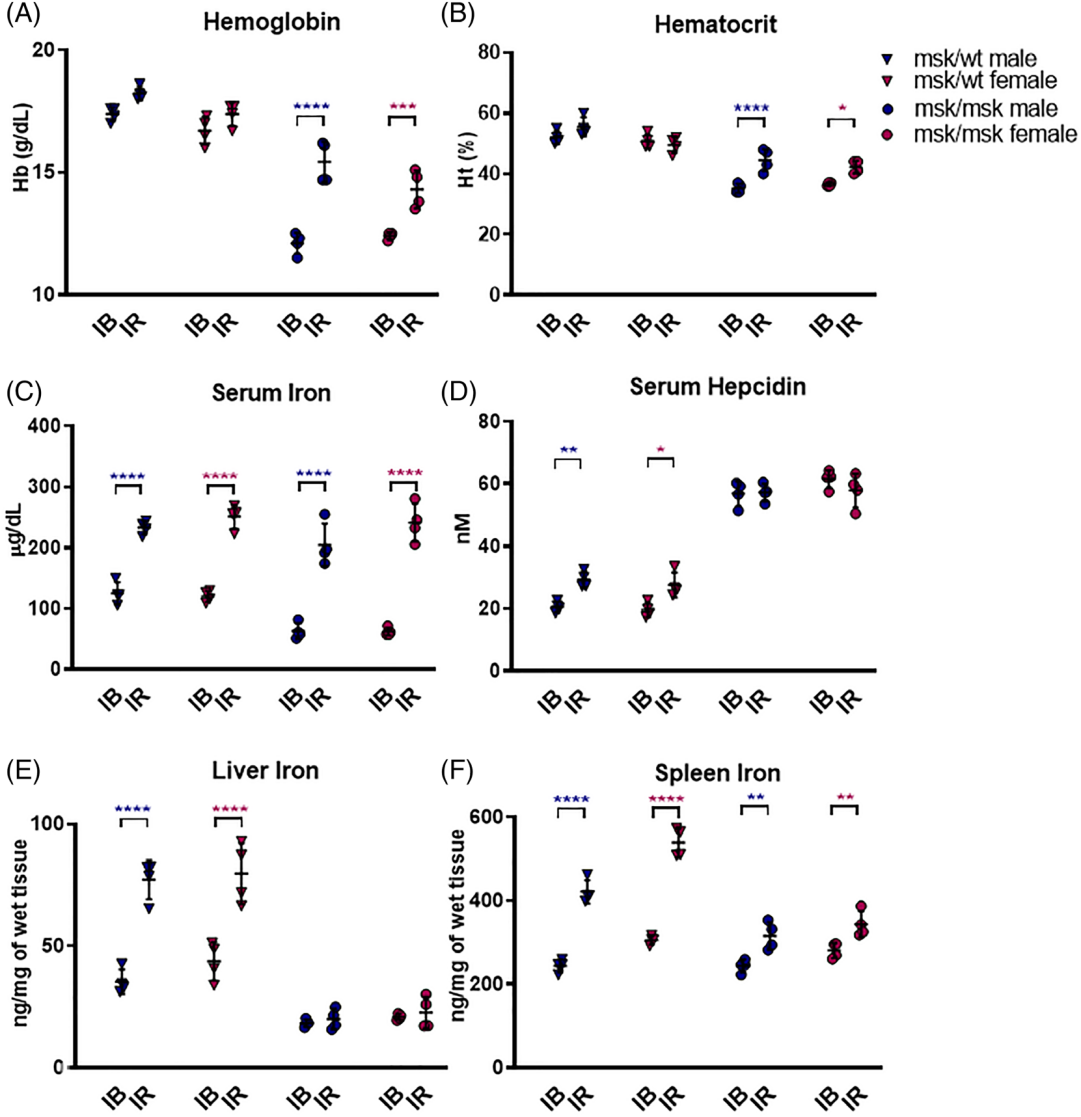
Iron and hematological parameters in msk/msk and msk/wt female and male mice (9‐week‐old) after 10 days of iron rich diet. (A) Hemoglobin (Hb) and (B) Hematocrit (Ht) levels measured using Hemo_Vet instrument; (C) Serum Iron was measured using commercial kit; (D) Hepcidin protein in serum was measured by SELDI‐TOF. (E) Liver and (F) Spleen iron content was detected spectrophotometrically. The female mice were marked in pink whereas male in blue (for both: circle was used for msk/msk, and inverted triangle for msk/wt). Each group consisted of four animals. Statistical analysis comparison between: female (pink asterisks), male (blue asterisks) in IB versus IR (respectively Iron balance diet vs. Iron rich diet), as indicated by the black line. ****p < 0.0001, ***p < 0.001, **p < 0.01, *p < 0.05 [Color figure can be viewed at wileyonlinelibrary.com]

Confirming the already published data, we fed 9‐week‐old mice (both msk/wt and msk/msk) with an iron‐rich diet (8.3 g/kg carbonyl iron) for 10 days. In both female and male msk/wt mice, the iron‐rich diet did not increase Hb and Ht level (Figure 2(A),(B)) but caused an increase of serum iron (from 122.67 ± 13.75 to 242.24 ± 17.84 μg/dl) (Figure 2(C)) and its accumulation in liver (from 39.48 ± 7.56 to 78.36 ± 9.84 ng/mg) and spleen (from 274.42 ± 34.91 to 480.00 ± 69.25 ng/mg) (Figure 2(E),(F)), as expected. Serum hepcidin and its liver mRNA increased in msk/wt mice (Figure 2(D) and S3A), as a consequence of increased iron stores (Figure 2(E),(F)) without signs of inflammation (Figure S3(C),(E)). However, after the iron‐rich diet, msk/msk mice showed increased Hb level from 12.25 ± 0.35 to 14.86 ± 0.96 g/dl and increased Ht with a similar trend (Figure 2(A),(B)), in both sexes confirming the results from other studies. Serum iron increased from 62.61 ± 9.63 to 223.06 ± 36.38 μg/dl both in females and males (Figure 2(C)), while liver iron was not changed and spleen iron level showed a little but significant increase (Figure 2(E),(F)). The serum and liver mRNA hepcidin levels were not modified (Figure 2(D) and S3(A)) and there were no signs of inflammation (Figure S3(C),(E)). In msk/msk mice the iron‐rich diet showed an increased iron in the brain of female mice and mainly in the duodenum in both sexes (Figure S4(A),(B)), as well in gastrocnemius and vastus lateralis (Figure S4(E),(F)), while no changes were observed in heart and kidney (Figure S4(C),(D)).

**FIGURE 3:**
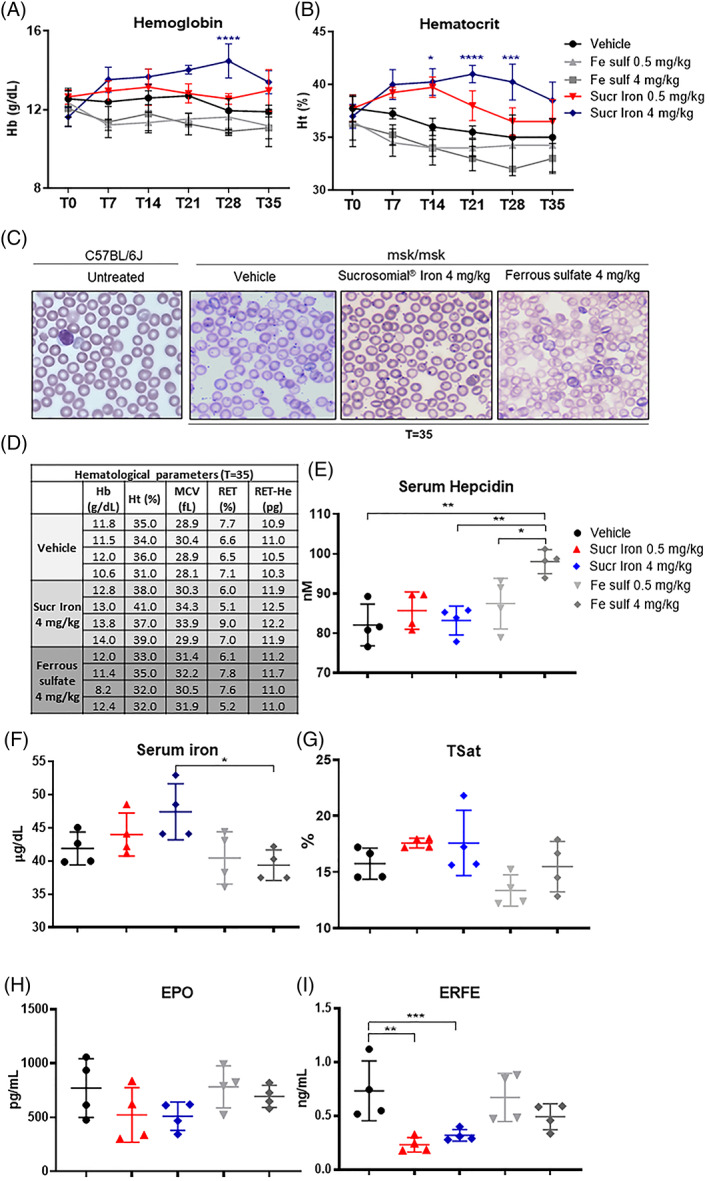
Hematological parameters in homozygous female mice (9‐week‐old) treated with Ferrous sulfate and Sucrosomial iron for 35 days. (A), Hemoglobin (Hb) and (B) Hematocrit (Ht) levels measured during the treatments (at days 0, 7, 14, 21, 28 and 35), using Hemo_Vet instrument. Statistical analysis in (A) and (B): the blue asteriks are the comparison between vehicle and Sucrosomial iron (4 mg/kg). At the end of treatments, (C) representative images of peripheral blood smear (May–Grünwald staining) of untreated C57BL/6J mice (as healthy control) and treated mice (Vehicle, Sucrosomial iron, ferrous Sulfate) at the end of the treatments (T = 35). (D) Level of Hemoglobin (Hb), Hematocrit (Ht), MCV, RET and Ret‐He in msk/msk mice at the end of the treatments. (E) Hepcidin protein in serum was measured by SELDI‐TOF; (F) Serum iron and (G) TSat were measured using commercial kit; (H) EPO and I, ERFE in the serum were measured using commercial ELISA kit (R&D and Intrinsic respectively). Each group consisted of four animals. Statistical analysis was done comparing the vehicle group versus treated ones, as indicated by the black line and asterisks. ****p < 0.0001, ***p < 0.001, **p < 0.01, *p < 0.05 [Color figure can be viewed at wileyonlinelibrary.com]

Then, we analyzed two oral iron formulations, ferrous sulfate and Sucrosomial iron, already largely used in humans, administering them in Mask mice as exactly the same amount of elemental iron. We choose female mice since, even if we did not observe any significant differences between the two sexes, the females presented a more constant level of Hb than males, between the 9 and 28 weeks of age, the period selected for the iron administration (females from 12.80 ± 0.80 to 13.80 ± 0.90 g/dl vs. males from 11.50 ± 0.90 to 14.20 ± 0.90 g/dl) (Table S1). The two iron formulations were administered by daily oral gavage at the doses of 0.5 and 4 mg/kg (of elemental iron) for 35 days and Hb and Ht were measured every week (T0, T7, T14, T21, T28 and T35). The Sucrosomial iron treatment caused an increase of Hb and Ht (Figure 3(A),(B)) particularly evident with 4 mg/kg. Thus, Hb increased from 11.50 ± 0.60 to 13.30 ± 0.8 g/dl at day 35 (Figure 3(A)), and Ht from 34.00 ± 1.80 to 38.80 ± 2.70% (Figure 3(B)). Vehicle did not affect Hb levels, while Ht showed a steady decline. Surprisingly during the treatment with ferrous sulfate, we observed a decrease of Hb levels, albeit minor, and a more evident decrease of Ht, at both doses even if at the end of the experiment (T35) the levels were comparable to the vehicle group (Figure 3(A),(B),(D)). The Hb and Ht values at the end of the treatments (T35), were significantly increased only in Sucrosomial iron group as well as a little increase was observed in the reticulocytes Hb concentration (RET‐He), whereas no significant differences were found in MCV and reticulocytes (RET) (Figure 3(D)). The blood smears stained with May–Grünwald showed that the Mask mice treated with vehicle and ferrous sulfate displayed cells with a central pallor and very low intense staining, whereas after Sucrosomial iron, the erythrocytes appeared normochromic compared to that of C57BL/6J mice (Figure 3(C)). This correlates with the observed data where vehicle and ferrous sulfate treated mice remained anemic, while in Sucrosomial iron treated mice the level of hemoglobin increased from 11.50 ± 0.60 to 13.30 ± 0.80 g/dl.

The groups treated with ferrous sulfate showed an increase of hepcidin (Figures 3(E) and S3(B)) that is statistically significant in serum hepcidin level with the dose of 4 mg/kg (Figure 3(E)). The hepcidin increase is probably due to an inflammatory status, as showed with a higher level of liver Socs3 and Saa1 mRNA, compared to the other treated groups (Figure S3(D)–(F)). No increase of liver hepcidin expression was found in the first 18 h after treatment with both formulations of oral iron (not shown). In Sucrosomial iron groups, the level of serum EPO was not strongly affected even if the level of ERFE was significantly reduced (Figure 3(H),(I)).

**FIGURE 4:**
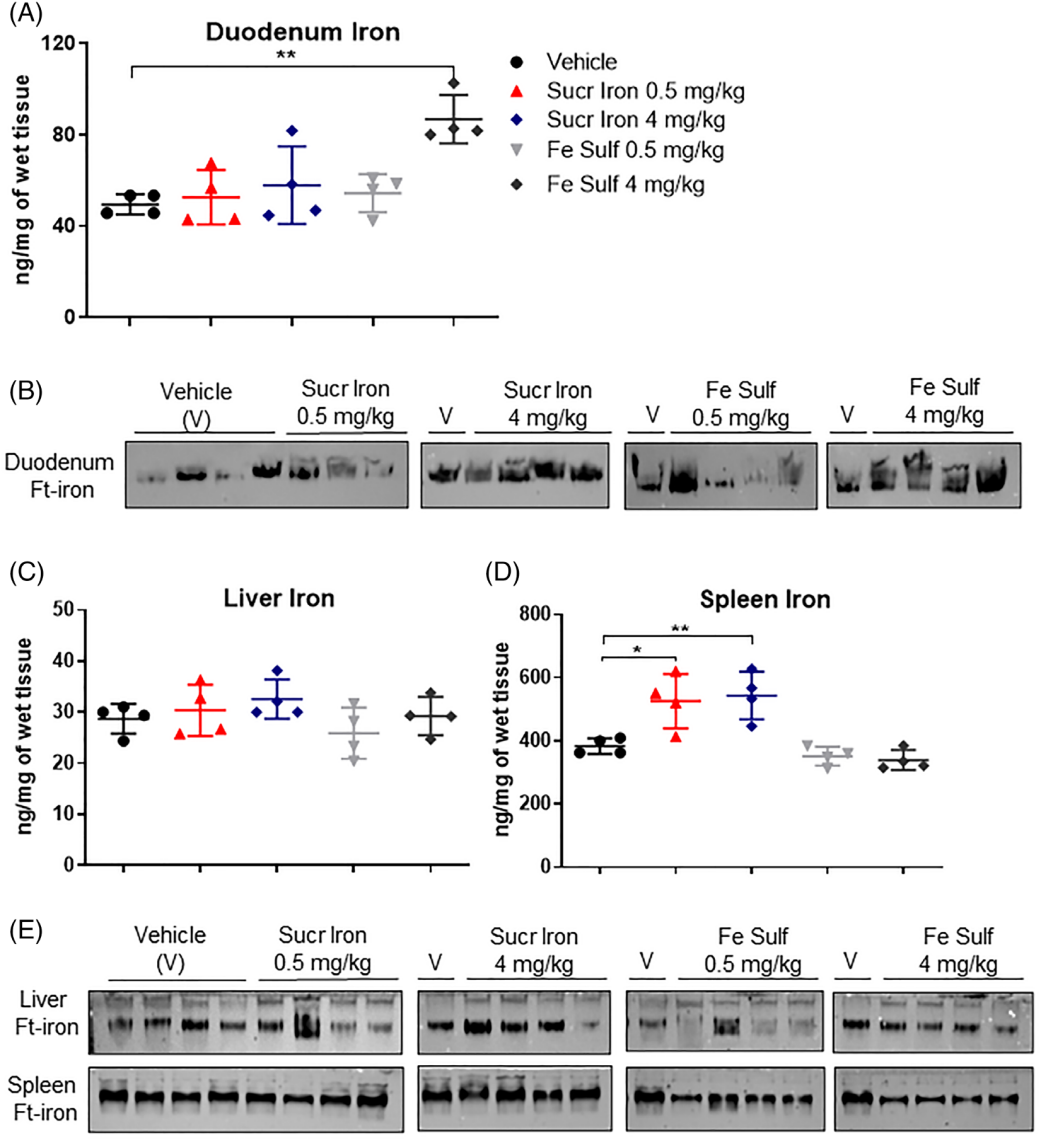
Analysis of duodenum, liver and spleen in msk/msk female mice (9‐week‐old) treated with ferrous sulfate and Sucrosomial iron for 35 days. At the end of experiment (day 35): (A), (C), (D) Duodenum, liver and spleen iron content was analyzed respectively. Statistical analysis was done comparing the vehicle group versus treated ones, as indicated by the black line and asterisk. **p < 0.01, *p < 0.05; (B) and (E) Ferritin‐iron (Ft‐iron) in duodenum liver and spleen was detected by Prussian blue staining plus DAB enhancement. Each group consisted of four animals [Color figure can be viewed at wileyonlinelibrary.com]

Serum iron showed higher values only in some mice treated with Sucrosomial iron (mainly with 4 mg/kg) compared to the vehicle or ferrous sulfate treated groups (Figure 3(F)). The TSat seems not to be affected in all the treated groups with some variability inside the groups (Figure 3(G)). The treatment with 0.5 mg/kg of ferrous sulfate did not induce an important iron accumulation in the tissues analyzed both as total iron (Figures 4(A),(C),(D) and S7, inverted gray triangles) and ferritin iron (Figure 4(B),(E)). Interestingly, the 4 mg/kg of ferrous sulfate induced a significant iron accumulation in duodenum both as total iron (Figure 4(A) dark gray diamonds) and ferritin iron (Figure 4(E)). The treatment with both 0.5 and 4 mg/kg of Sucrosomial iron increased the total iron in spleen (Figure 4(D)) and brain (Figure S7(A)) and no significant changes in the other tissues analyzed (Figure S7), whereas only the highest dose induced a minor increase of ferritin iron in all the tissues analyzed more evident in duodenum and liver (Figure 4(B),(E)).

Hematoxilin and eosin staining of duodenum samples showed no differences in the morphology of intestinal villi from mice treated with vehicles or with Sucrosomial iron and ferrous sulfate at both concentrations (0.5 and 4 mg/kg), with no visible areas of erosion and atrophy (Figure S6). The treatment with 0.5 mg/kg of both Sucrosomial iron and ferrous sulfate induced only a slight iron accumulation in duodenal villi compared with that observed in vehicles treated mice as assessed with Prussian blue staining (Figure S6). The treatment with 4 mg/kg of Sucrosomial iron induced an increase of iron accumulation in duodenal villi compared to low dose of Sucrosomial iron but less than that in the duodenum of mice treated with 4 mg/kg of ferrous sulfate (Figure S6).

Immunohistochemical analysis for CD3 positive intraepithelial lymphocytes showed an increase of these cells in duodenal villi in mice treated with both Sucrosomial iron and ferrous sulfate compared to the vehicles (Figure S6) without substantial differences between the two iron formulations.

Interestingly, the alopecia, evident in all Mask mice before the treatments, was not changed in the vehicle‐treated animals and even less in the ferrous sulfate mice, while a substantial recover was observed in the Sucrosomial iron group (Figure S5(A)–(C), T = 0 vs. T = 35).

As a further analysis, we put the control msk/wt female mice on an iron‐deficient (ID) diet for 6 weeks to make them anemic (Hb levels comparable to those of the Mask homozygous mice, 11.76 ± 0.82 g/dl msk/wt after ID diet vs. 11.50 ± 0.60 msk/msk). This reduced both liver mRNA and serum hepcidin level, as expected (comparing mice in IB with ID diet) (Figure S8(G),(H)), without sign of inflammation (Figure S9(E),(F)). Then, they were treated by oral‐gavage with Sucrosomial iron and ferrous sulfate, as above in presence of ID diet for all the time of the experiment. The Hb and Ht levels recovered to normal levels after 28 days of treatment with both formulations (Figure S8(A),(B)). Due to the recovery of hemoglobin level, EPO and ERFE were strongly reduced after the treatments with both the formulations of oral iron (Figure S8(E),(F)).

The two formulations caused an increase of serum iron, Tsat (Figure S8(C),(D)), liver and spleen iron content (Figure S9(B),(C)); the increase is more evident in the highest doses. Ferrous sulfate at 4 mg/kg caused a major increase of duodenum iron (Figure S9A) similar to that observed in the Mask msk/msk mice (Figure 4(A)) suggesting an iron retention. In the ferrous sulfate treated groups, we observed an increase of hepcidin (Figure S8(G),(H)) and of two markers of inflammation, liver Socs3 and Saa1 (Figure S9(E),(F); observed also in msk/msk mice, Figure S3(D),(F)).

## 4 DISCUSSION

So, IRIDA is an autosomal recessive rare disease caused by non‐functional MT2, that is unable to cut HJV and suppress the hepatic BMP6/SMAD signaling, leading to a constantly expression of high levels of hepcidin and consequently FPN degradation, with impaired iron absorption from the gut and reduced iron stores in the body. Besides increased hepcidin levels, the main features of IRIDA typically include moderate/severe microcytic anemia, low transferrin saturation and paradoxically normal to slightly increased ferritin levels, without signs of inflammation. Because of refractoriness to oral iron, the current treatment in IRIDA syndrome is based on parenteral iron supplementation, although its efficacy is often incomplete, requiring repeated lifelong administrations.^19^, ^20^, ^21^

The Mask mouse^16^ displays two‐to‐four‐fold higher hepcidin levels than normal, and represents a valuable model of human IRIDA, allowing to study the mechanisms of iron absorption and distribution as well as the effects of novel therapeutic approaches.

We analyzed homozygous Mask (msk/msk) mice in comparison with heterozygous (msk/wt) mice, as controls, to characterize their systemic iron status. Mask mice had lower serum iron levels and lower iron content in different organs across all age groups and in both sexes, confirming the essential role of hepcidin as the regulator of systemic iron homeostasis. Accordingly, they showed lower Hb levels (12.19 ± 1.64 g/dl) compared to heterozygous (16.41 ± 1.92 g/dl), a difference evident just before the weaning and that persisted during aging in both sexes. In particular, we observed more severe anemia and reduced amount of liver and spleen iron content in male Mask mice than in females, although their hepcidin levels were comparable. Moreover, we found decreased muscle iron content in the Mask mice compared to controls, in accordance with a previous study.^38^ The Mask mice showed reduced iron assimilation, altered muscle metabolism, and they stopped growing after 1 week and lost body fat. The phenotype was rescued by IV administration of high‐dose iron dextran, showing that muscle iron deficiency was the main cause. Interestingly, the Mask mice were smaller than the msk/wt of the same age (Table S1). All the differences were maintained during the ages analyzed (3,6,9,15 and 28 weeks). Therefore, this model offers also an opportunity to study the role of iron on muscle status and performance, as it was done in a TfR1 KO mouse model.^39^

As expected, in the Mask model, the duodenum was the only tissue in which iron content was higher than in controls, as a consequence of the hepcidin‐mediated FPN degradation. On the contrary, no iron accumulation has been detected in spleen, suggesting that anemia in IRIDA mice cannot be solved by mobilizing iron, but rather by overcoming the block of intestinal iron absorption, through FPN‐independent mechanisms.

We confirmed that the Mask mice treated with an iron‐rich diet, increased Hb and serum iron levels indicating that carbonyl iron could be absorbed even in high hepcidin expression status, probably bypassing the hepcidin‐ferroportin axis block. Thus, it could be an effective iron formulation to solve the anemia in IRIDA patients, but more studies are needed and the actual mechanism of its absorption remains to be elucidated.

In the present study, we mainly compared the same amount of elemental iron of two different oral iron formulations: ferrous sulfate in the form of ionic iron (Fe^2+^) and pyrophosphate iron in the form of sucrosomial particles (Sucrosomial iron). The histological evaluation of duodenum did not show evident differences between the two iron formulations both in terms of duodenal villi morphology and CD3^+^ infiltrated lymphocytes apart from an increase of iron accumulation in duodenal villi in mice treated with 4 mg/kg of ferrous sulfate. It is known that iron supplementation can induce alterations in jejunum,^40^ in the colon and in the gut microbiota^41^, ^42^ but it is still an open field of research and it requires more studies. In this context, it will be important to evaluate, in a dedicated study, different sections of the intestinal tract such as jejunum^40^ as well as microbiota in Mask mice before and after iron supplementation to better understand this mouse model, the effect of different iron formulations and the reason why we found an alteration of inflammatory markers in the liver of mice treated with ferrous sulfate.

Primarly, we found that ferrous sulfate is ineffective in curing the anemia of Mask mice, suggesting it is mainly absorbed via FPN‐dependent mechanism, which requires hepcidin suppression that typically occurs under iron deficiency conditions. In fact, it solved the anemia of the iron deficient mice, even if it induced an inflammatory response, confirming previous observations by our group,^30^ possibly due to its higher duodenal retention, or due to an alteration along the gastrointestinal tracts.

Interestingly, Sucrosomial iron improved Hb and Ht in the control mice without inflammatory side effects. Moreover, only the Sucrosomial iron increased Hb, Ht, serum and spleen iron concentration in Mask mice in 35 days without signs of inflammation. Although its mechanism of absorption is not fully clarified, the results suggest that it could actually involves FPN‐independent pathway. This is in agreement with a recent case report of a young patient affected by IRIDA, in which the authors showed that Sucrosomial iron treatment led to a persistent increase of Hb levels.^22^

Overall, our data suggest the Sucrosomial iron efficacy in the IRIDA mouse model, characterized by high hepcidin levels that hamper absorption of traditional oral iron formulations, such as ferrous sulfate. This encourages to study in detail the mechanisms of its absorption in mice, an important step to promote its use in clinical practice in hepcidin‐driven anemias.

## CONFLICT OF INTERESTS

Elisa Brilli and Germano Tarantino are PhatmaNutra S.p.a. employees. All the other authors declare no conflict of interest.

## AUTHOR CONTRIBUTIONS

Michela Asperti planned the experiments, performed the research and analyzed data. Elisa Brilli planned the experiments, analyzed data. Andrea Denardo and Magdalena Gryzik performed the research. Francesca Pagani performed the histological analysis. Germano Tarantino planned the experiments and contributed to write the paper. Fabiana Busti, Paolo Arosio, Domenico Girelli contributed to writing the paper. Maura Poli planned the experiments, analyzed data and wrote the paper.

## Supporting information


**Table S1** Mean levels of Hemoglobin (Hb), Hematocrit (Ht) and weight of C57BL/6J wild‐type, heterozygous (msk/wt) and homozygous (msk/msk) Mask mice during the age.

**Table S2** Mean levels of Red Blood Cells (RBC), Mean Corpuscular Volume (MCV), Reticulocytes (RET) and Reticulocytes‐Hemoglobin (Ret‐He) of heterozygous (msk/wt) and homozygous (msk/msk) Mask mice, both male (upper table) and female (lower table) during the age.

**Appendix S1:** Supplementary Information.

## Data Availability

Data available on request from the authors
