# Supplementary material for: Iron distribution in different tissues of homozygous Mask (msk/msk) mice and the effects of oral iron treatments
Source: Am J Hematol. 2021 Aug 14;96(10):1253–63. doi: 10.1002/ajh.26311 (PMC9292262; doi:10.1002/ajh.26311)
Supplement: Supplementary file 1 — Table S1 Mean levels of Hemoglobin (Hb), Hematocrit (Ht) and weight of C57BL/6J wild‐type, heterozygous (msk/wt) and homozygous (msk/msk) Mask mice during the age. [file AJH-96-1253-s001.doc]

**Supplemental Table1**

| **Male C57/BL/6J wild-type versus msk/wt and msk/msk** | | | | | |
| --- | --- | --- | --- | --- | --- |
| **Hb (g/dL)** | **3 weeks** | **6 weeks** | **9 weeks** | **15 weeks** | **28 weeks** |
| wild-type | 14.3 ± 0.8 | 15.5 ± 0.6 | 15.9 ± 1.1 | 16.2 ± 0.9 | 16.7 ± 0.5 |
| msk/wt | 13.0 ± 0.9 | 15.9 ± 1.0 | 16.2 ± 0.7 | 16.3 ± 0.5 | 16.9 ± 0.9 |
| msk/msk | 11.0 ± 0.6 | 9.8 ± 1.0 | 11.5 ± 0.9 | 11.6 ± 0.5 | 14.2 ± 0.9 |
| **Ht (%)** | **3 weeks** | **6 weeks** | **9 weeks** | **15 weeks** | **28 weeks** |
| wild-type | 39.0 ± 0.5 | 46.0 ± 0.9 | 45.4 ± 0.8 | 49.2 ± 1.0 | 50.3 ± 1.5 |
| msk/wt | 40.0 ± 2.6 | 47.0 ± 2.9 | 48 ± 1.8 | 50.0 ± 1.3 | 51.0 ± 0.6 |
| msk/msk | 32.3 ± 1.7 | 29.0 ± 2.7 | 34.0 ± 2.4 | 34.0 ± 1.4 | 41.5 ± 2.5 |
| **Weight (g)** | **3 weeks** | **6 weeks** | **9 weeks** | **15 weeks** | **28 weeks** |
| wild-type | 15.0 ± 1.9 | 20.1 ± 1.4 | 26.5 ± 1.3 | 29.5 ± 1.3 | 33.3 ± 2.9 |
| msk/wt | 15.4 ± 2.4 | 21.6 ± 1.2 | 26.1 ± 1.1 | 30.1 ± 1.7 | 33.1 ± 2.0 |
| msk/msk | 10.9 ± 1.2 | 15.7 ± 1.3 | 22.1 ± 1.9 | 24.8 ± 0.9 | 26.9 ± 1.8 |

| **Female C57/BL/6J wild-type versus msk/wt and msk/msk** | | | | | |
| --- | --- | --- | --- | --- | --- |
| **Hb (g/dL)** | **3 weeks** | **6 weeks** | **9 weeks** | **15 weeks** | **28 weeks** |
| wild-type | 13.3 ± 0.5 | 16.4 ± 0.7 | 16.8 ± 0.9 | 16.9 ± 0.5 | 17.1 ± 0.9 |
| msk/wt | 13.7 ± 0.7 | 16.7 ± 0.8 | 16.9 ± 1.1 | 17.1 ± 1.1 | 17.3 ± 0.5 |
| msk/msk | 10.9 ± 0.6 | 12.3 ± 1.0 | 12.9 ± 0.8 | 14.5 ± 1.1 | 13.8 ± 0.9 |
| **Ht (%)** | **3 weeks** | **6 weeks** | **9 weeks** | **15 weeks** | **28 weeks** |
| wild-type | 41.2 ± 2.2 | 43.5 ± 2.1 | 46.0 ± 3.4 | 49.7 ± 1.6 | 51.0 ± 3.1 |
| msk/wt | 39.9 ± 6.4 | 45.3 ± 2.5 | 46.5 ± 3.5 | 50.9 ± 2.9 | 51.0 ± 1.4 |
| msk/msk | 32.3 ± 1.5 | 36.0 ± 2.9 | 37.8 ± 2.5 | 42.5 ± 3.3 | 40.5 ± 2.6 |
| **Weight (g)** | **3 weeks** | **6 weeks** | **9 weeks** | **15 weeks** | **28 weeks** |
| wild-type | 11.6 ± 1.5 | 16.5 ± 0.7 | 20.3 ± 0.9 | 21.5 ± 1.2 | 23.6 ± 0.7 |
| msk/wt | 11.0 ± 1.4 | 16.0 ± 0.9 | 20.7 ± 0.5 | 22.4 ± 1.2 | 23.8 ± 0.4 |
| msk/msk | 10.0 ± 0.7 | 15.1 ± 0.8 | 18.8 ± 0.4 | 21.3 ± 0.6 | 22.0 ± 1.4 |

**Supplemental Table1:** Mean levels of Hemoglobin (Hb), Hematocrit (Ht) and weight of C57BL/6J wild-type, heterozygous (msk/wt) and homozygous (msk/msk) Mask mice during the age
