# Supplementary material for: Iron distribution in different tissues of homozygous Mask (msk/msk) mice and the effects of oral iron treatments
Source: Am J Hematol. 2021 Aug 14;96(10):1253–63. doi: 10.1002/ajh.26311 (PMC9292262; doi:10.1002/ajh.26311)
Supplement: Supplementary file 2 — Table S2 Mean levels of Red Blood Cells (RBC), Mean Corpuscular Volume (MCV), Reticulocytes (RET) and Reticulocytes‐Hemoglobin (Ret‐He) of heterozygous (msk/wt) and homozygous (msk/msk) Mask mice, both male (upper table) and female (lower table) during the age. [file AJH-96-1253-s002.doc]

**Supplemental Table2**

| **Male msk/wt versus msk/msk** | | | | |
| --- | --- | --- | --- | --- |
| **RBC (M/𝛍L)** | **3 weeks** | **9 weeks** | **15 weeks** | **28 weeks** |
| msk/wt | 4.59 ± 0.83 | 9.09 ± 0.93 | 9.52 ± 0.34 | 7.88 ± 2.04 |
| msk/msk | 3.89 ± 0.98 | 11.76 ± 0.27 | 12.59 ± 0.59 | 10.61 ± 0.68 |
| **MCV (fL)** | **3 weeks** | **9 weeks** | **15 weeks** | **28 weeks** |
| msk/wt | 45.27 ± 2.14 | 44.55 ± 0.77 | 46.03 ± 0.81 | 46.70 ± 0.91 |
| msk/msk | 38.75 ± 1.77 | 28.43 ± 1.01 | 27.70 ± 0.12 | 29.08 ± 0.96 |
| **RET (%)** | **3 weeks** | **9 weeks** | **15 weeks** | **28 weeks** |
| msk/wt | 17.73 ± 1.18 | 4.28 ± 0.36 | 4.60 ± 0.76 | 4.66 ± 0.79 |
| msk/msk | 22.74 ± 1.66 | 6.06 ± 1.03 | 5.36 ± 0.43 | 6.98 ± 0.57 |
| **Ret-He (pg)** | **3 weeks** | **9 weeks** | **15 weeks** | **28 weeks** |
| msk/wt | 14.30 ± 1.04 | 17.38 ± 0.17 | 17.40 ± 0.10 | 17.15 ± 0.29 |
| msk/msk | 11.55 ± 2.05 | 10.53 ± 0.80 | 10.85 ± 0.13 | 10.68 ± 0.33 |

| **Female msk/wt versus msk/msk** | | | | |
| --- | --- | --- | --- | --- |
| **RBC (M/𝛍L)** | **3 weeks** | **9 weeks** | **15 weeks** | **28 weeks** |
| msk/wt | 2.56 ± 0.30 | 9.23 ± 0.16 | 9.66 ± 0.04 | 10.12 ± 0.27 |
| msk/msk | 4.86 ± 0.13 | 11.94 ± 0.64 | 11.94 ± 0.27 | 11.30 ± 0.62 |
| **MCV (fL)** | **3 weeks** | **9 weeks** | **15 weeks** | **28 weeks** |
| msk/wt | 44.15 ± 2.33 | 44.95 ± 0.92 | 43.77 ± 0.15 | 41.67 ± 0.42 |
| msk/msk | 38.15 ± 0.64 | 28.50 ± 0.84 | 30.08 ± 1.03 | 30.33 ± 1.24 |
| **RET (%)** | **3 weeks** | **9 weeks** | **15 weeks** | **28 weeks** |
| msk/wt | 19.49 ± 4.71 | 4.29 ± 0.28 | 3.46 ± 0.27 | 3.49 ± 0.21 |
| msk/msk | 21.83 ± 3.86 | 5.27 ± 0.52 | 4.31 ± 0.35 | 6.08 ± 0.48 |
| **Ret-He (pg)** | **3 weeks** | **9 weeks** | **15 weeks** | **28 weeks** |
| msk/wt | 13.95 ± 2.47 | 17.85 ± 0.07 | 17.30 ± 0.17 | 17.0 ± 0.44 |
| msk/msk | 11.50 ± 0.71 | 11.38 ± 0.70 | 12.35 ± 0.58 | 11.20 ± 0.76 |

**Supplemental Table2:** Mean levels of Red Blood Cells (RBC), Mean Corpuscular Volume (MCV), Reticulocytes (RET) and Reticulocytes-Hemoglobin (Ret-He) of heterozygous (msk/wt) and homozygous (msk/msk) Mask mice, both male (upper table) and female (lower table) during the age
