# Supplementary material for: Iron distribution in different tissues of homozygous Mask (msk/msk) mice and the effects of oral iron treatments
Source: Am J Hematol. 2021 Aug 14;96(10):1253–63. doi: 10.1002/ajh.26311 (PMC9292262; doi:10.1002/ajh.26311)
Supplement: Supplementary file 3 — Appendix S1: Supplementary Information. [file AJH-96-1253-s003.doc]

**SUPPORTING INFORMATION**

*May–Grünwald Giemsa staining.* Peripheral blood smears were stained with May-Grünwald (MG) Giemsa (Sigma Aldrich) for morphological examination. Briefly, blood smears were incubated with pure MG 1 minute at room temperature, followed 5 minutes incubation with 50% MG diluted in distilled water. After washing in tap water, blood smears were incubated 20 minutes at room temperature with 20% Giemsa diluted in distilled water. Smears were washed with tap water, dried and mounted with xylene. Images were acquired with Nikon DS-Ri2 camera (4908x3264 full-pixel) mounted on Nikon Eclipse 50i microscope equipped with Nikon Plan lenses using NIS-Elements imaging software 4.3 (Nikon Corporation). 60X original magnification.

*Quantitative qRT-PCR.* Total RNA was isolated from tissues using TRIzol Reagent (Ambion), according to the manufacturer’s instruction. cDNA was generated by Reverse transcription, using 1 µg RNA and Improm-II Reverse Transcriptase (Promega) in 20 µL and analyzed by quantitative reverse transcription polymerase chain reaction (qRT-PCR), using PowerUp SYBR Green Master Mix (Life Technologies) according to the manufacturer’s instructions. All data were normalized to the expression of Hprt1 and expressed as Relative Quantification (method of 2^−ΔΔCt).

The primers used are:

MmHprt1: For 5-CTGGTTAAGCAGTACAGCCCCAA-3, Rev 5-CAGGAGGTCCTTTTCACCAGC-3;

MmHep: For 5-AAGCAGGGCAGACATTGCGAT-3, Rev 5-CAGGATGTGGCTCTAGGCTATGT-3;

MmSocs3: For 5-TTAAATGCCCTCTGTCCCAGG-3, Rev 5-TGTTTGGCTCCTTGTGTGCC-3;

MmSaa1 For 5-AGAGGACATGAGGACACCAT-3; Rev 5-CAGGAGGTCTGTAGTAATTGG-3;

MmId1: For 5-ACCCTGAACGGCGAGATCA-3, Rev 5- TCGTCGGCTGGAACACATG-3.

*Iron quantification.* Tissues iron content was determined spectrophotometrically as previously described^1^. Briefly, 50 mg of wet tissue was incubated for 18 h at 65°C in 0.5 mL of 3 M HCl and 0.6 M trichloroacetic acid. After centrifugation, 10 µL of sample was added to 240 µL of working chromogen reagent, containing 1 vol of 0.1% bathophenanthroline sulfonate/1% thioglycolic acid solution, 5 vol of water, and 5 vol of saturated sodium acetate, in a 96 well plate. The samples were then incubated for 30 min at room temperature and the absorbance measured at 535 nm in a plate reader. A standard curve was prepared with a pre-calibrated solution of FeCl_3_ (Sigma-Aldrich). The obtained value was normalized for the mg of tissue initially used for this analysis.

*Ferritin Iron evaluation*. Duodenum, liver and spleen homogenates were heated at 70°C for 10 min to enrich ferritins. Samples (equivalent to 200 µg for duodenum and 100 µg for liver and spleen of preheated protein) were loaded on 7.5% non-denaturing PAGE and run for 3 h at 160 V. The gels were washed with water and incubated in 2% ferrocyanide (Sigma-Aldrich) and 2% HCl for 1 h. To enhance the signal, the gels were incubated in 0.025% 3,30-diaminobenzidine (Sigma-Aldrich) and 0.05% H_2_O_2_ in 1X TBE for 15–60 min. The reaction was stopped by washing with tap water.

*Hepcidin, Erythropoietin (EPO), Erythroferrone (ERFE) and Iron in the serum.* Mouse serum hepcidin was quantified using a validated mass-spectrometry based assay ^2,3^, recently updated ^1,4,5^. EPO and ERFE in the serum were quantified using commercial Elisa Kits (Cod.MEP00B from R&D and Cod.ERF-200 from Intrinsic Lifesciences respectively). Serum iron was determined spectrophotometrically with a commercial kit, according to the manufacturer’s instruction (Cod. SI257 from Randox Laboratories).

*Histochemistry and immunostaining on paraffin-embedded duodenal sections.* Duodenal samples were formalin-fixed and paraffin-embedded. Representative sections were selected based on adequate tissue preservation, as assayed by hematoxylin and eosin (H&E) staining.

De-waxed tissue sections were stained with Perls Prussian blue stain for non-heme iron by using standard procedure. Briefly, sections were de-waxed, re-hydrated and rinsed in running water and PBS. The slides were then submerged in 8% ferrocyanide, 5% HCl for 25 min, rinsed with water and counterstained with nuclear red solution. Immunostaining for CD3 was performed on 2-μm-thick paraffin sections using a rabbit monoclonal anti-CD3 antibody (SP7 clone; Abcam; 1:100). Sections were de-waxed, re-hydrated and endogenous peroxidase activity blocked with 0.3% H_2_O_2_ in methanol for 20 minutes. Antigen retrieval was performed using a microwave-oven in 1.0 mM EDTA buffer (pH 8.0). Sections were then washed in TBS (pH 7.4) and incubated for one hour in the primary antibody diluted in TBS 1% bovine serum albumin. The reaction was revealed by using Novolink Polymer (Leica Microsystems) followed by DAB and slides counterstained with Hematoxylin. Images were acquired with Nikon DS-Ri2 camera (4908x3264 full-pixel) mounted on Nikon Eclipse 50i microscope equipped with Nikon Plan lenses using NIS-Elements imaging software 4.3 (Nikon Corporation). Scale bar: 40X and 60X original magnification, corresponding to 50µm and 20 µm respectively.

**SUPPORTING FIGURES**

**FIGURE S1**


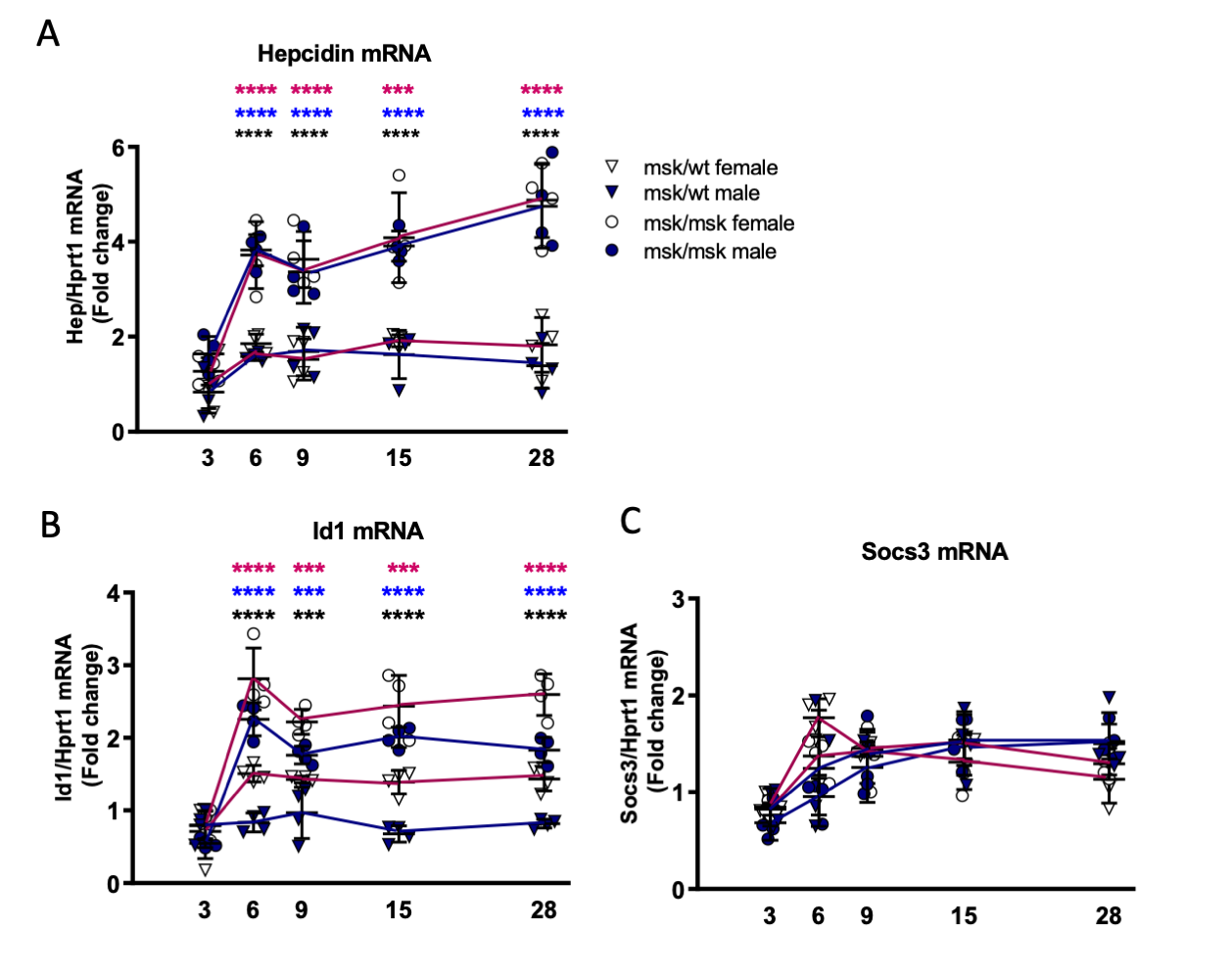


**FIGURE S1. Hepcidin,** Id1 and Socs3 in msk/msk and msk/wt female and male mice in different ages. (A) Hepcidin, (B) Id1 and (C) Socs3 mRNA levels from liver measured by qPCR and normalized for Hprt1. The female mice were marked with pink line and empty circles (for homozygous, msk/msk) or inverted triangles (for heterozygous, msk/wt), whereas male in blue line and blue circles (for homozygous, msk/msk) or inverted triangles (for heterozygous, msk/wt). Each group consisted of 4 animals. Statistical analysis: * comparison between msk/msk and msk/wt female; * between msk/msk and msk/wt female; * between msk/msk and msk/wt mixed sexes, for each age. ****P<0.0001, ***P<0.001

**FIGURE S2**

**
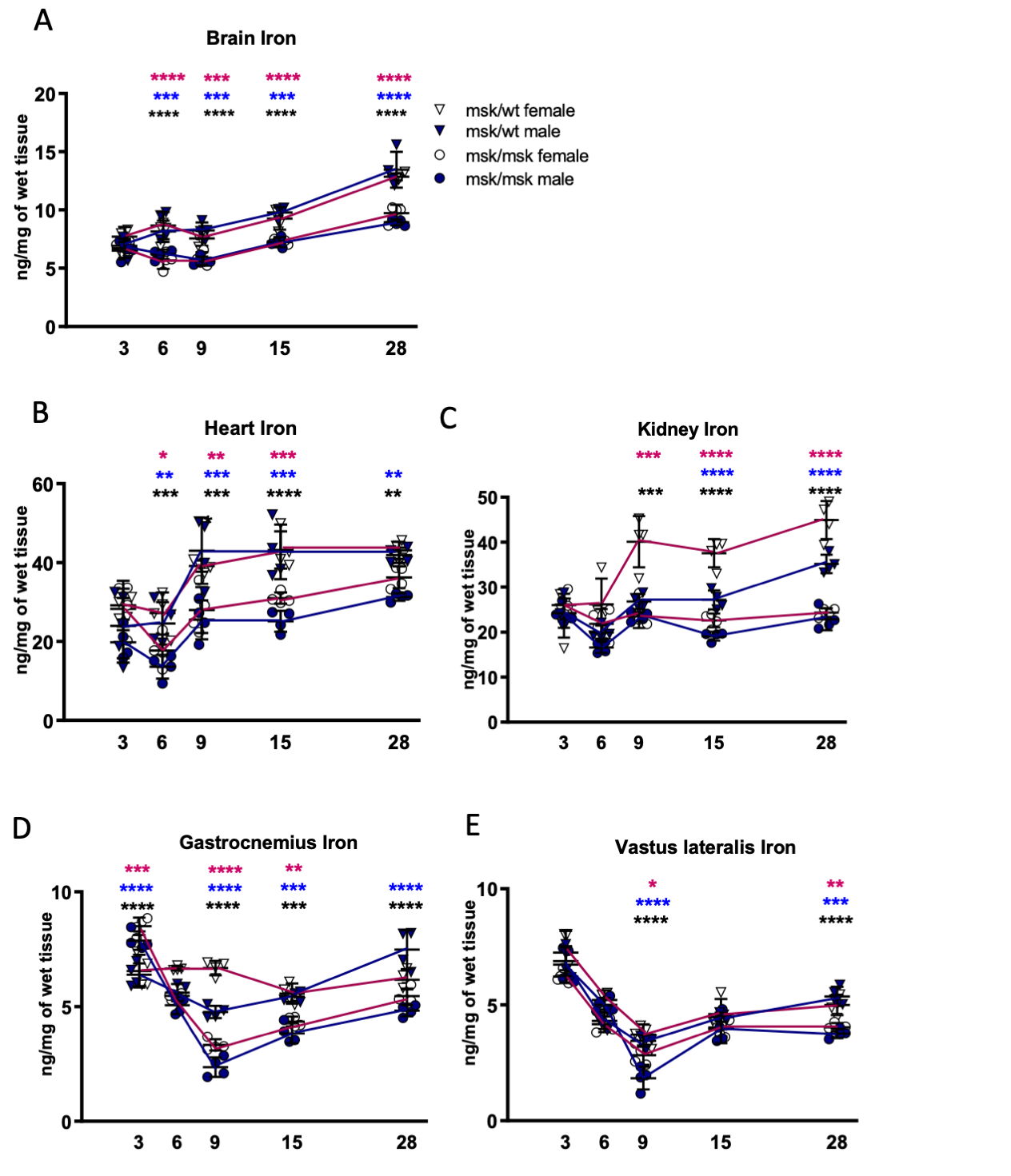
**

**FIGURE S2.** Iron content in msk/msk and msk/wt female and male mice in different organs. Iron in the (A) Brain, (B) Heart, (C) Kidney, (D) Gastrocnemius and (E) Vastus Lateralis muscles was spectrophotometrically detected in each age analysed (3-6-9-15-28 weeks). The female mice were marked with pink line and empty circles (for homozygous, msk/msk) or inverted triangles (for heterozygous, msk/wt), whereas male in blue line and blue circles (for homozygous, msk/msk) or inverted blue triangles (for heterozygous, msk/wt). Each group consisted of 4 animals. Statistical analysis, comparison between: * msk/msk and msk/wt female; * msk/msk and msk/wt male; * msk/msk and msk/wt mixed sexes, for each age. ****P<0.0001, ***P<0.001, **P<0.01, *P<0.05

**FIGURE S3**


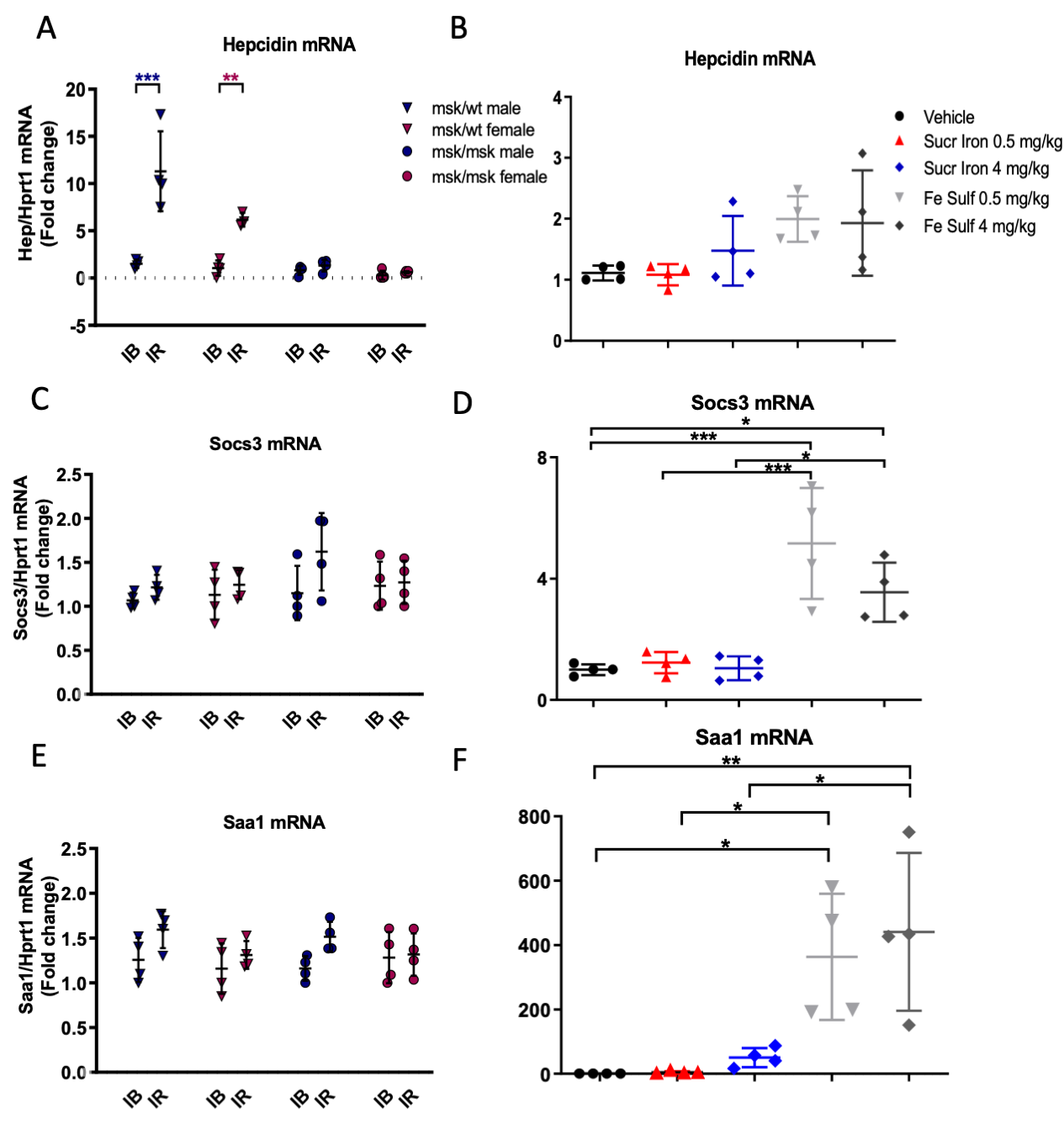


**FIGURE S3. Hepcidin,** Socs3 and Saa1 in msk/msk and msk/wt female and male mice after (9-week-old) maintained in iron rich diet for 10 days or msk/msk female mice (9-week-old) treated with Ferrous sulfate and Sucrosomial® Iron for 35 days. (A-B) Hepcidin, (C-D) Socs3 and (E-F) Saa1 mRNA levels from liver measured by qPCR and normalized for Hprt1. Each group consisted of 4 animals. In (A-C-E) the female mice were marked in pink whereas male in blue (for both: circle was used for msk/msk, and inverted triangle for msk/wt). Statistical analysis comparison between: * female; * male in IB vs IR (respectively Iron Balance diet versus Iron Rich diet), as indicated by the black line. In (B-D-F) statistical analysis was done comparing the vehicle group versus treated ones, as indicated by the black line and asterisks. ***P<0.001, **P<0.01, *P<0.05

**FIGURE S4**


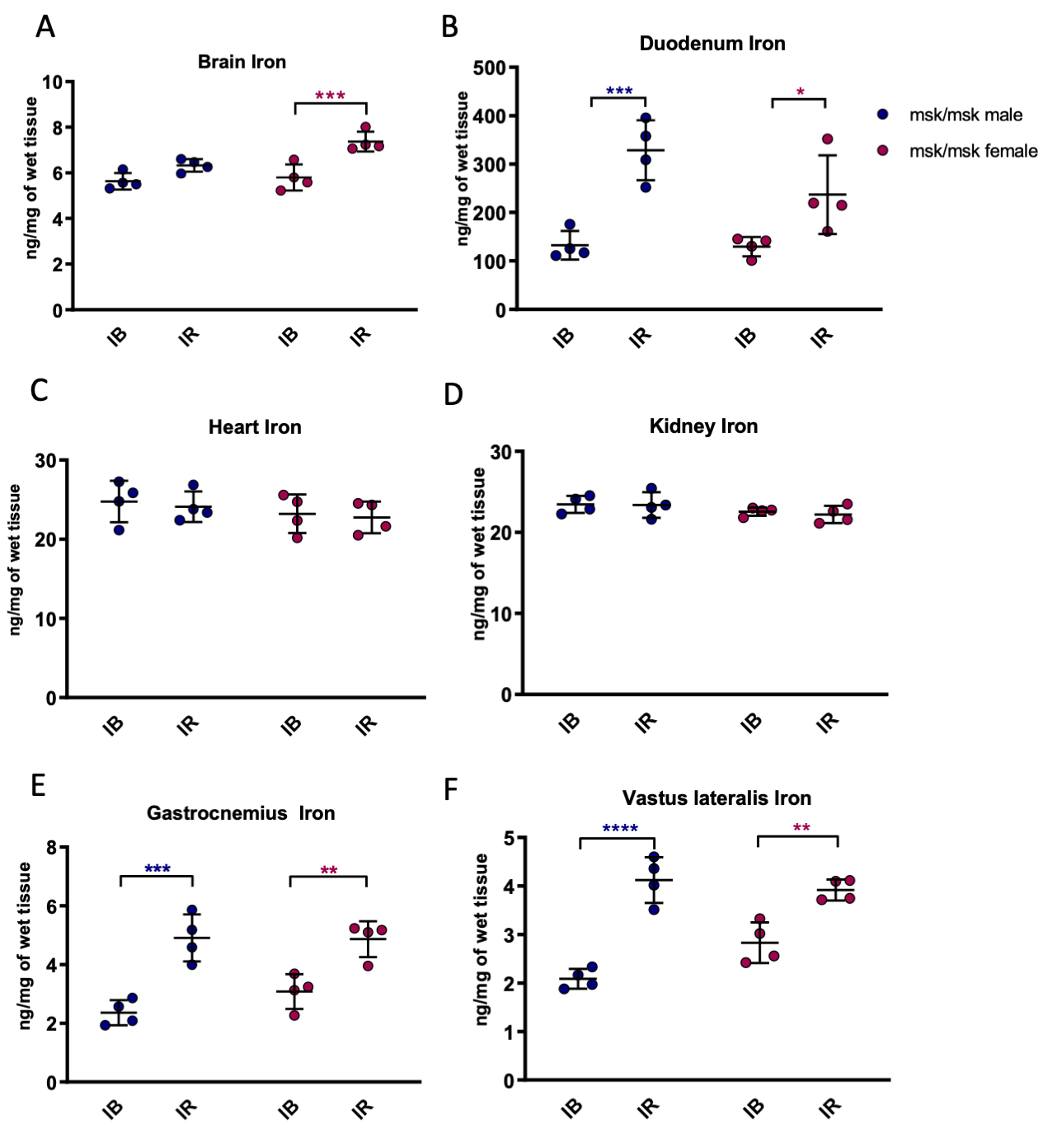


**FIGURE S4.** Iron content in different organs in msk/msk male and female mice (9-week-old) maintained in iron rich diet for 10 days. (A) Brain, (B) Duodenum, (C) Heart, (D) Kidney, (E) Grastrocnemius and (F) Vastus lateralis iron content was spectrophotometrically detected. Each group consisted of 4 animals. The female mice were marked in pink whereas male in blue. Statistical analysis was done comparing the vehicle group versus treated ones, as indicated by the black line and asterisk. ****P<0.0001, ***P<0.001, **P<0.01, *P<0.05

**FIGURE S5**


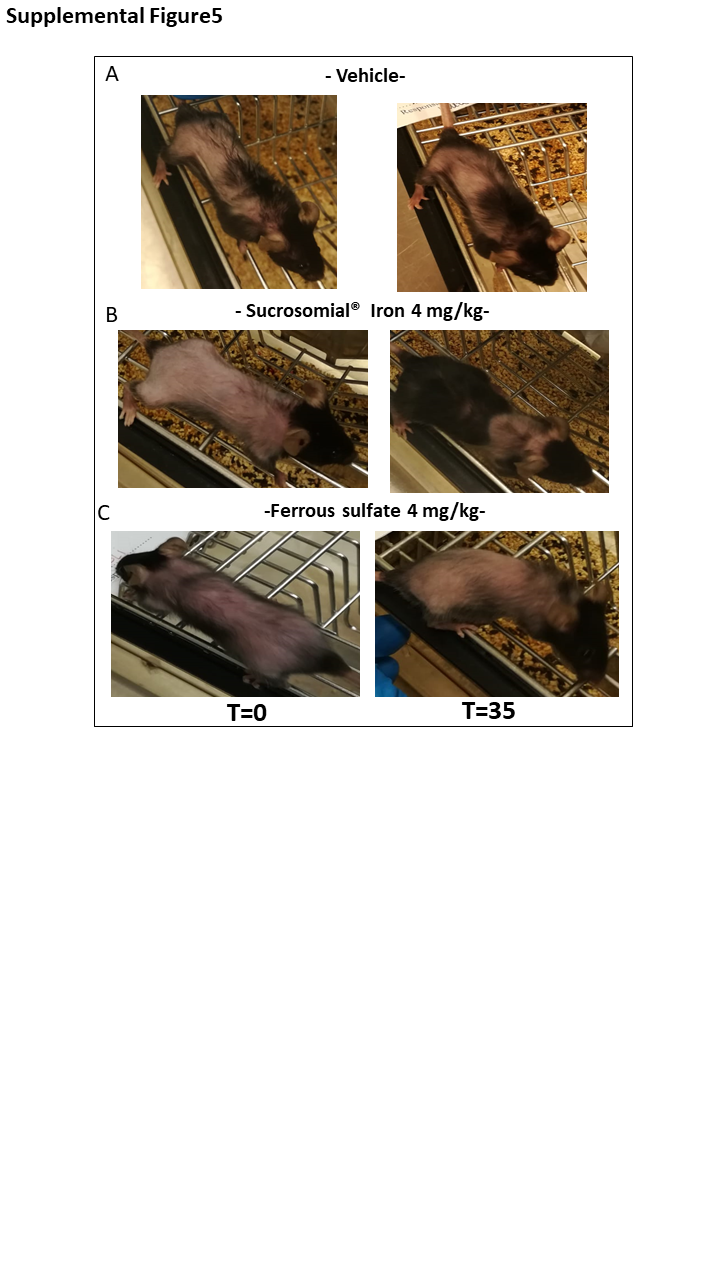


**FIGURE S5.** Representative images of the body hair of msk/msk mice at day 0 (T=0) and after 35 days (T=35) of treatment with (A) vehicle, (B) Sucrosomial® Iron 4 mg/kg and (C) Ferrous sulfate 4 mg/kg.

**FIGURE S6**

**
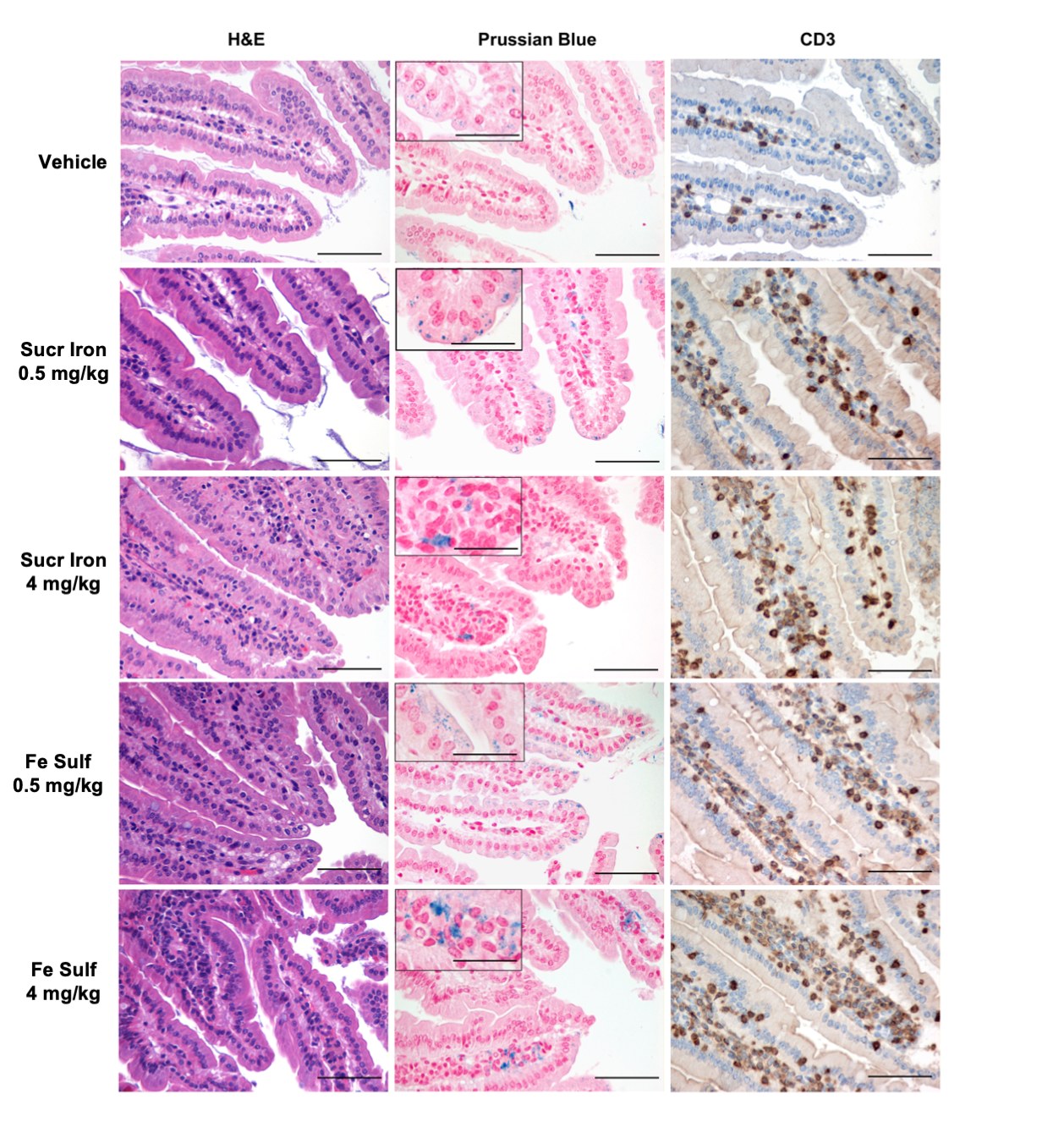
**

**FIGURE S6.** Representative images (40X) of duodenum stained with H&E, Prussian Blue and CD3. Images were acquired with Nikon DS-Ri2 camera (4908x3264 full-pixel) mounted on Nikon Eclipse 50i microscope equipped with Nikon Plan lenses using NIS-Elements imaging software 4.3 (Nikon Corporation). Scale bar: 40X and 60X original magnification, corresponding to 50µm and 20 µm respectively.

**Figure S7**

**
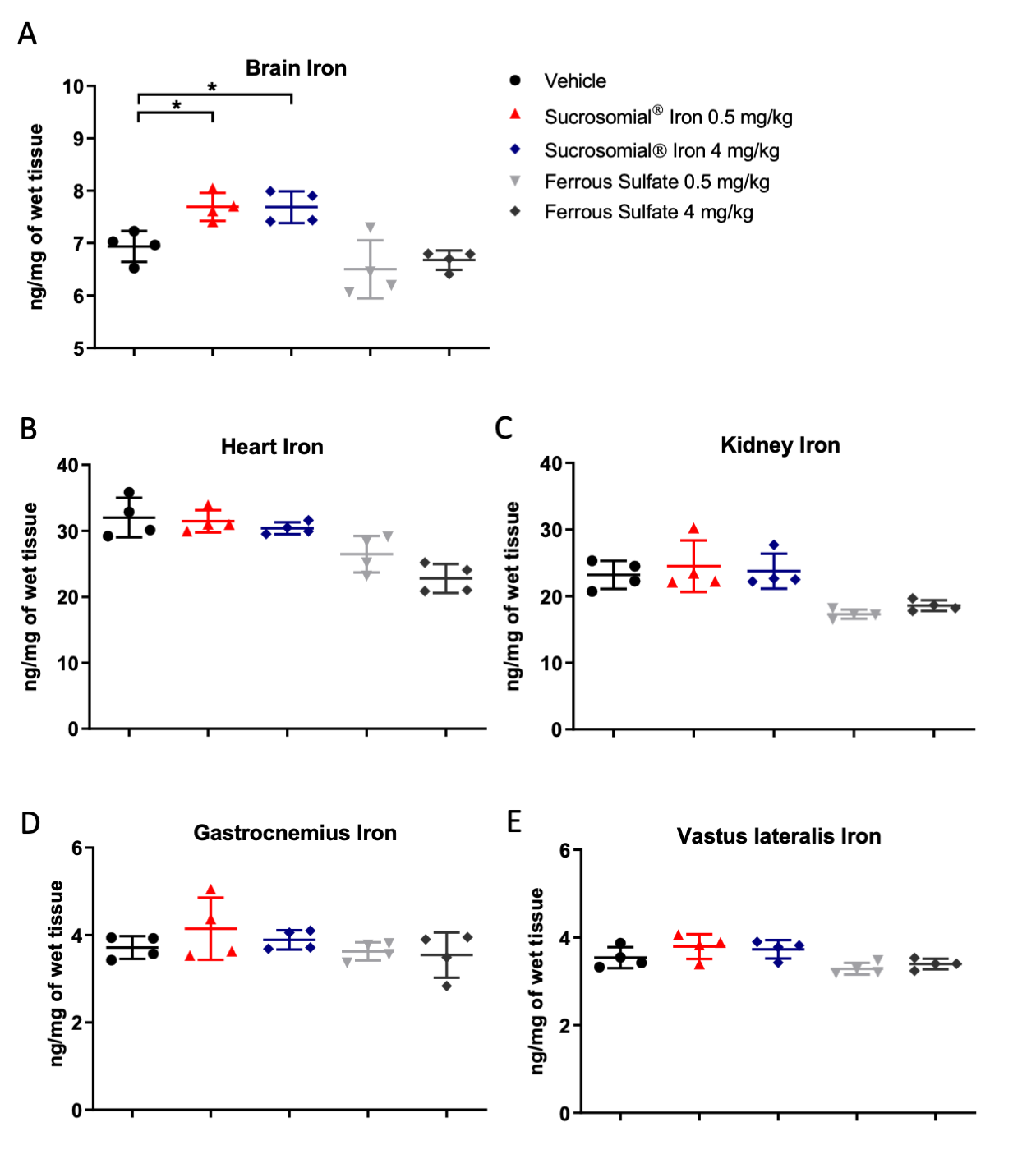
**

**FIGURE S7** Iron content in different organs in msk/msk female mice (9-week-old) treated with Ferrous sulfate and Sucrosomial® Iron for 35 days. At the end of experiment (day 35): (A) Brain, (B) Heart, (C) Kidney, (D) Gastrocnemius and (E) Vastus lateralis iron content was spectrophotometrically detected. Each group consisted of 4 animals. Statistical analysis was done comparing the vehicle group versus treated ones, as indicated by the black line and asterisk. **P<0.01, *P<0.05.

**FIGURE S8**

**
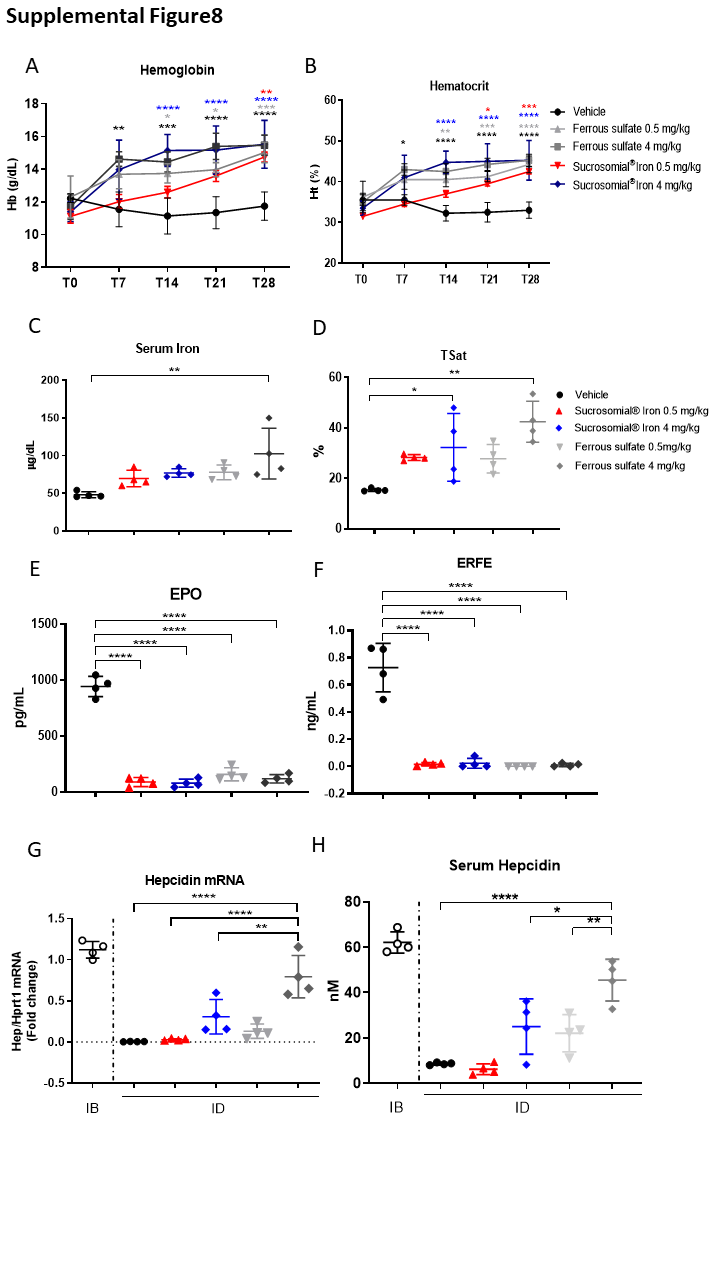
**

**FIGURE S8.** Iron and hematological parameters in msk/wt female mice (9-week-old) made anemic with iron free diet and thentreated with Ferrous sulfate and Sucrosomial® iron for 28 days. (A) Hemoglobin and (B) Hematocrit levels measured during the treatments (at days 0, 7, 14, 21 and 28), using Hemo_Vet instrument. Statistical analysis in (A) and (B): * comparison between vehicle and Sucrosomial® Iron female (0.5 mg/kg); * between vehicle and Sucrosomial® iron female (4 mg/kg); * between vehicle and Ferrous sulfate (0.5 mg/kg); * between vehicle and Ferrous sulfate (4 mg/kg). At the end of experiment (Day 28) (C) Serum iron and (D) Transferrin saturation (TSat) were detected by commercial kit. (E) EPO and (F) ERFE in the serum were measured using commercial ELISA kit (R&D and Intrinsic respectively); (G) Hepcidin mRNA in the liver was measured by qPCR and normalized for Hprt1. (H) Hepcidin protein in the serum was measured by SELDI-TOF. Each group consisted of 4 animals. Statistical analysis was done comparing the vehicle group versus treated ones, when the differences were statistically significant, they were indicated by the black line and asterisks. IB= Iron balance diet; ID= Iron deficiency diet. ****P<0.0001, ***P<0.001, **P<0.01, *P<0.05

**FIGURE S9**


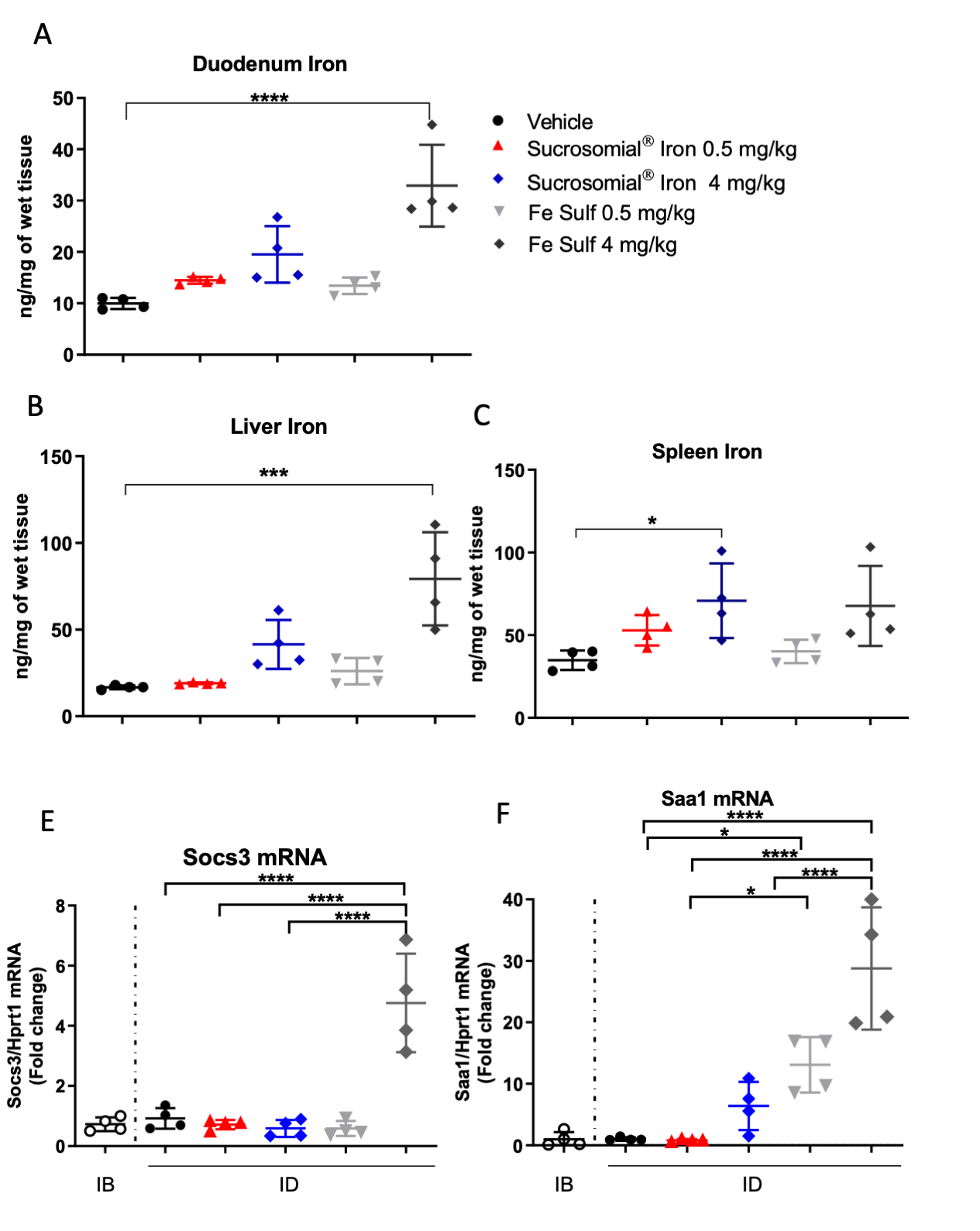


**FIGURE S9.** Iron content, Socs3 and Saa1 mRNA in msk/wt female mice (9-week-old) treated with Ferrous sulfate and Sucrosomial® Iron for 28 days. At the end of experiment (T28): (A) Duodenum, (B) Liver and (C) Spleen iron content were spectrophotometrically detected. (E) Socs3 and Saa1 mRNA in the liver was measured by qPCR and normalized for Hprt1. Each group consisted of 4 animals. Statistical analysis was done comparing the vehicle group versus treated ones, as indicated by the black line and asterisks. ****P<0.0001, *P<0.05

**REFERENCES**

1. Asperti M, Gryzik M, Brilli E, et al. Sucrosomial. *Nutrients*. 2018;10(10).

2. Castagna A, Campostrini N, Zaninotto F, Girelli D. Hepcidin assay in serum by SELDI-TOF-MS and other approaches. *J Proteomics*. 2010;73(3):527-536.

3. Poli M, Asperti M, Naggi A, et al. Glycol-split nonanticoagulant heparins are inhibitors of hepcidin expression in vitro and in vivo. Blood. Vol. 123. United States; 2014:1564-1573.

4. Girelli D, Nemeth E, Swinkels DW. Hepcidin in the diagnosis of iron disorders. *Blood*. 2016;127(23):2809-2813.

5. Udali S, Castagna A, Corbella M, et al. Hepcidin and DNA promoter methylation in hepatocellular carcinoma. *Eur J Clin Invest*. 2018;48(2).
